# Hepatobiliary complications of immune checkpoint inhibitors in cancer

**DOI:** 10.37349/etat.2024.00257

**Published:** 2024-07-26

**Authors:** Donna Zhuang, David Zhang, Stephen Riordan

**Affiliations:** University of Salford, UK; ^1^Gastrointestinal and Liver Unit, Prince of Wales Hospital, Randwick, New South Wales 2031, Australia; ^2^Faculty of Medicine and Health, University of New South Wales, Randwick, New South Wales 2031, Australia

**Keywords:** Immune checkpoint, immune checkpoint inhibitors, hepatobiliary, hepatitis, cholangitis, immune-related adverse events

## Abstract

Immune checkpoint inhibitors (ICIs) have dramatically changed the landscape of cancer therapy. Over the last decade, both their primary focus in trials and clinical application have exponentially risen, with repeated demonstrations of their efficacy in improving survival in various cancer types. The adverse effects of these drugs on various organ systems were recognised in early phase studies. Given their relatively new emergence on the market, there has been increasing interest into short- and long-term effects and management of ICIs in real-world settings. ICI-related hepatobiliary toxicities are often challenging to diagnose and difficult to distinguish from other causes of deranged liver biochemical tests. The aim of this review is to provide an up-to-date and detailed exploration of the hepatobiliary complications of ICIs, including pathogenesis and approaches to diagnosis and management.

## Introduction

Immune checkpoint inhibitors (ICIs) are a rapidly growing cornerstone of cancer therapy that has revolutionised the treatment paradigm and improved the overall prognosis of patients with cancer. Since the breakthrough approval of ipilimumab in 2011 for metastatic melanoma, more than 5,000 clinical trials have been conducted to investigate the role of ICIs in more than 15 types of cancer [1]. The most studied ICI types are cytotoxic T-lymphocyte-associated 4 (CTLA-4) inhibitors, programmed cell death protein-1 (PD-1) inhibitors, and programmed death ligand-1 (PD-L1) inhibitors. Due to increased ICI use, there is an increasing awareness of organ-specific immune-related adverse events and their potential clinical impact. Moreover, certain patient populations, such as those with pre-existing autoimmune diseases, immunocompromised patients, and the elderly were excluded from initial clinical trials but are now increasingly being considered for ICI use in mainstream clinical practice [2–5]. Understanding the pathophysiology of ICI-related adverse events and identifying populations at increased risk are important to help guide both appropriate patient selection for these treatments and the implementation of appropriate monitoring strategies.

This review aims to summarise the current evidence surrounding the incidence, pathogenesis, clinical manifestations, diagnosis, management, and prognosis of hepatobiliary complications of ICIs. A relevant literature search was conducted with the following electronic databases: Ovid MEDLINE, PubMed Central, EMBASE, and Google Scholar. Several search terms were used with keywords as fields and free texts, as well as in different permutations; examples include “immune checkpoint inhibitors adverse effects”, “immune checkpoint” AND “hepatobiliary”, and “immune checkpoint inhibitors adverse” AND (“hepatic” OR “biliary”). All studies published between the years 2011 and 2024 were considered and screened by their title, abstract, and keywords.

## Definition and terminology

The exact definition of ICI-related hepatobiliary injury is heterogenous amongst clinical trials and guidelines, although definitions employed are based on liver biochemical tests and whether a pattern of derangement is consistent with a hepatocellular or cholestatic picture. The grading of liver injury in the majority of ICI studies is in accordance with the National Cancer Institute’s Common Terminology Criteria for Adverse Events (CTCAE) v5.0 published in November 2017, as summarised in Table 1.

**Table 1:** Grading of liver biochemical test derangement

**Liver enzyme**	**Baseline level**	**Grade 1**	**Grade 2**	**Grade 3**	**Grade 4**
Alanine aminotransferase (ALT) and aspartate aminotransferase (AST)	If baseline normal	> ULN to 3× ULN	> 3–5× ULN	> 5–20× ULN	> 20× ULN
	If baseline abnormal	1.5–3× baseline	> 3–5× baseline	> 5–20× baseline	> 20× baseline
Alkaline phosphatase (ALP)	If baseline normal	> ULN to 2.5× ULN	> 2.5–5× ULN	> 5–20× ULN	> 20× ULN
	If baseline abnormal	2–2.5× baseline	> 2.5–5× baseline	> 5–20× baseline	> 20× baseline
Bilirubin	If baseline normal	> ULN to 1.5× ULN	> 1.5–3× ULN	> 3–10× ULN	> 10× ULN
	If baseline abnormal	> 1–1.5× baseline	> 1.5–3× baseline	> 3–10× baseline	> 10× baseline

ULN: upper limit of normal

The International Consortium for Innovation and Quality in Pharmaceutical Development developed the encompassing term encompassing term, “immune-mediated liver injury caused by checkpoint inhibitors (ILICI)” in 2020, with hepatocellular [predominant elevations in alanine aminotransferase (ALT) and aspartate aminotransferase (AST)], cholestatic [predominant elevation in alkaline phosphatase (ALP)], and mixed (similar degrees of elevation of hepatocellular-type and cholestatic-type liver enzymes) variants of ILICI are recognised [6].

## Incidence and risk factors

Nearly all published studies use deranged liver biochemistry to identify and classify patients with ILICI [7]. A histopathology study of 27 patients with ILICI revealed that more than half (52%) of the liver injury patterns seen in ILICI are hepatocellular, whilst cholestatic (19%) and mixed hepatocellular and cholestatic (29%) patterns substantially less common [8]. This study analysed the correlation between liver biochemistry values and these three histological patterns of liver injury seen in ILICI and found that peak ALT values were significantly higher in patients with the hepatocellular pattern of histological liver injury than in patients with cholestatic liver injury, resulting in a good discriminative capacity to classify patients as having hepatocellular-type ILICI. Peak ALP values were significantly lower in the hepatocellular-type ILICI group compared with those in patients with cholestatic or mixed hepatocellular and cholestatic histological appearances, resulting in a good discriminative capacity to classify patients as having cholestatic- or mixed hepatocellular and cholestatic-type ILICI [8]. When classified by liver biochemistry rather than on histological grounds in a larger cohort of 117 patients with ILICI, the prevalence of hepatocellular-type and cholestatic-type ILICI were found to be similar (38% and 37%, respectively), with the remaining 25% of patients conforming to a mixed hepatocellular and cholestatic-type pattern [9].

AST and ALT elevations attributed to ICI use occur varyingly at an incidence of 3–20% with single ICI treatment regimens [10–15]. Grades 3 or 4 ILICI have an incidence of 0.5–15% [12, 16, 17].

A higher incidence and grade of severity of ILICI may be associated with exposure to CTLA-4 inhibitors compared to PD-1 and PD-L1 inhibitors [17–21]. Moreover, there seems to be a dose-dependent relationship between ICI exposure and the risk of developing ILICI [11, 22]. The incidence of ILICI also increases with combination therapy in the form of dual ICIs, an ICI with chemotherapy, or an ICI with other biological therapy [10, 13, 14, 20, 21, 23–27]. A meta-analysis of more than 2,000 patients found that dual ICI therapy had a relative risk of 2.54 for all grade ILICI, as well as a relative risk of 2.7 for high-grade ILICI, compared to monotherapy [28]. Hepatic failure related to ICI use has been reported but is rare [20, 23, 29, 30]. A systematic review and network meta-analysis suggested that the rate of fatal liver adverse events related to ILICI is in the order of 0.07% [20]. Data from the US Food and Drug Administration Adverse Reporting System database indicate that the median time from ICI initiation to hepatic failure onset was relatively short at 38 days and that over 68% of patients died after developing hepatic failure. The data suggested that PD-L1 inhibitors may carry an increased risk for hepatic failure compared to PD-1 inhibitors and CTLA-4 inhibitors [23].

Incidence of liver enzyme elevations encountered during ICI therapy was found in a territory-wide cohort study in Hong Kong (China) to be increased in patients undergoing ICI treatment for hepatocellular carcinoma (HCC) compared with that in patients undergoing treatment for other malignancies [12]. A recent multi-centre prospective study involving patients from 20 tertiary care centres across Europe, the USA and Asia also reported a significantly increased rate of liver enzyme derangement (both any grade and grades 3 or 4) associated with ICI therapy in patients with HCC compared to other advanced solid organ tumours [31]. Attribution of causality of the liver enzyme derangement to ILICI was based on the assessment of treating physicians at each participating centre and on the exclusion of alternative aetiologies, including viral infections, active alcohol use, autoimmune liver disease, drugs other than ICIs, and disease progression, although the potential for causes other than ILICI must always be kept in mind in HCC patients. Nonetheless, the occurrence of liver enzyme derangement attributed to ILICI in the HCC group did not increase the rate of hepatic functional decompensation compared to that in patients without apparent ILICI, while HCC patients with grades 1 or 2 liver enzyme derangement attributed to ILICI were found to have both significantly longer overall survival in time-adjusted analysis and a significantly higher objective response rate compared to patients without apparent ICI-related adverse events. On the other hand, neither overall survival nor the objective response rate differed significantly in patients with grades 3 or 4 liver enzyme derangement attributed to ILICI compared to those without apparent ICI-related adverse events [31]. Patients undergoing ICI therapy with hepatic metastasis from extra-hepatic primary malignancies do not seem to be at increased risk of developing ILICI [32]. Similarly, paraneoplastic syndromes are not predictive of increased ILICI risk [33].

Cancer patients with pre-existing autoimmune diseases are increasingly being considered as candidates for ICI despite their historical exclusion from clinical trials [34]. A meta-analysis found that there was a greater risk of developing both any grade and severe grade ICI-related adverse events in those with pre-existing autoimmune diseases compared to those without. Patients with autoimmune diseases tended to have an increased incidence of ICI-related adverse events involving the same organ system as those affected by autoimmunity [3]. However, small studies have shown that pre-existing autoimmune liver disease does not necessarily preclude treatment with PD-1 and PD-L1 inhibitors [35]. Additional larger-scale studies are required to further determine the risk of ILICI in this patient cohort. Close clinical and liver biochemical monitoring is prudent in this group.

Underlying metabolic dysfunction-associated fatty liver disease may increase the predisposition to developing ILICI [36, 37]. Other factors that may be associated with an increased risk of developing ILICI include male sex, a history of previous ICI treatment, and use of paracetamol [23, 38, 39]. Treatment with 3-hydroxy-3-methyl-glutaryl-coenzyme A (HMG-CoA) reductase inhibitors may also be associated with an increased risk of grades 3 or 4 events. There is conflicting evidence as to whether age under or over 65 years is associated with the risk of ILICI [23, 38, 40]. While these various factors may prove to be important in predisposing to ILICI, it must be acknowledged that data are currently limited.

Risk factors for the less common cholestatic variant of ILICI are not well defined. Pathologically, cholestatic-type ILICI related to biliary involvement is classified by the degree of involvement as either small bile duct, large bile duct, or mixed. Post-marketing studies have reported that the incidence of large bile duct variants of cholestatic-type ILICI ranges from 0.05% to 0.7%. The true incidence of the small bile duct variant is unclear, given that this is a histological diagnosis and liver biopsy for histopathology is required to establish this [41].

## Pathogenesis

ICIs target the interactions of CTLA-4 and PD-1 with their respective ligands, namely CD80/86 and PD-L1. Binding of CTLA-4 with CD80/86 and of PD-1 with PD-L1 results in inactivation and downregulation of T cell activity. The inhibition of CTLA-4 or PD-L1/PD-1 activates T cells and promotes anti-tumour function. Some of the anti-CTLA-4, anti-PD-1, and anti-PD-L1 agents currently available for clinical use are listed in Table 2 below.

**Table 2:** Examples of immune checkpoint inhibitor drugs and their targets

**Target**	**Drugs**
Cytotoxic T-lymphocyte-associated protein 4 (CTLA-4)	Ipilimumab, tremelimumab
Programmed cell death protein-1 (PD-1)	Nivolumab, pembrolizumab, cemiplimab, dostarlimab, retifanlimab, toripalimab
Programmed death ligand-1 (PD-L1)	Atezolizumab, avelumab, durvalumab

The exact mechanism of ILICI has not been fully characterised. The hepatic immune system is constantly exposed to significant amounts of harmless dietary and commensal antigens from the gastrointestinal tract via the portal circulation, requiring a state of immune tolerance [42]. Specialised antigen-presenting cells including Kupffer cells, hepatic stellate cells, dendritic cells, and liver sinusoidal endothelial cells play an important role in mediating immune tolerance. Conversely, mechanisms also exist to override immune tolerance in order to generate a response to pathogenic antigens, requiring a balance between immune responsiveness and immune tolerance. By blocking key modulatory pathways, ICIs can result in a loss of immune tolerance, thereby generating an inflammatory response. There is a higher rate of immune-related adverse events, including hepatitis, in patients undergoing combination therapy with CTLA-4 and PD-1 inhibitors, indicating a cumulative effect [21].

Several mechanisms are proposed to be important in leading to ILICI. These include the expansion of T helper cells such as Th1 and Th17 cells, resulting in an increased production of pro-inflammatory cytokines, activation of monocytes, and reduction in regulatory T cells, in particular, those that express fork head box p3 (FOXP3) [43–45]. It has been postulated that immunotherapy can also cause direct cytotoxicity through complement pathway activation, although this would not explain why the liver would be specifically affected [46]. Other proposed mechanisms include the adhesion of activated T cells to hepatic sinusoids, causing interaction with Kupffer cells and inducing the secretion of tumour necrosis factor α (TNF-α) and resultant hepatocyte injury [47]. Therapy-related loss of immune tolerance may be driven by epitope spreading, where damage to tumour cells from immunotherapy results in the release of antigenic material that is shared with normal cells, promoting a secondary immune response to self-antigens [45].

Liver histopathology of hepatocellular-type ILICI demonstrates heterogenous findings. Lobular hepatitis with mild periportal inflammation is the most common manifestation with predominant lymphocytic infiltration [8, 48–50]. A variable degree of neutrophilic, eosinophilic, and plasma cell infiltration may also be present, and centrilobular necrosis is often also seen. This histological appearance differs from that of autoimmune hepatitis unrelated to ICI exposure, in which a predominance of plasma cell infiltration is a diagnostic feature. Infiltration of CD3+ and CD8+ lymphocytes is a feature of ILICI, whilst infiltration of CD20+ and CD4+ cells is less of a feature in ILICI compared to findings in autoimmune hepatitis and other forms of drug-induced liver injury [49]. Histology may be able to distinguish between CTLA-4 inhibitor-induced hepatitis, in which there is often the presence of fibrin ring granulomas and central vein endotheliitis, and that related to PD-1 and PD-L1 inhibitor-induced hepatitis, in which more extensive lobular hepatitis is generally apparent [8, 11, 51].

Histopathology of the cholestatic form of ILICI is heterogenous and non-specific. The most common finding is bile duct injury in the absence of lobular involvement, as well as neutrophilic pericholangitis [52]. The small bile duct variant often demonstrates portal inflammation with mononuclear or mixed inflammatory cell infiltration and a predominance of CD4+ and CD8+ T cell infiltration, while the large bile duct variant demonstrates inflammatory infiltration with diffuse fibrosis of the extrahepatic bile ducts [53–55]. Other features may include the presence of intraepithelial lymphocytes, periportal necrosis, biliary-type interface activity, and cholangiolitis [52].

## Clinical manifestations and diagnosis

Whilst onset can occur at any point during treatment, ILICI is most often seen after 4 to 16 weeks of treatment [8, 10, 51, 56]. There may also be an ongoing hepatotoxic effect despite discontinuation of therapy, a phenomenon that occurs more often with PD-1 and PD-L1 inhibitor therapies compared to treatment with CTLA-4 inhibitors [8, 17].

ILICI often develops insidiously. Patients most commonly are asymptomatic at diagnosis but may present with a myriad of non-specific symptoms such as fatigue, nausea, and malaise. Serial liver biochemical test monitoring (including bilirubin, AST, ALT, and ALP values) is crucial for detection [13].

A diagnosis of ILICI involves the exclusion of other causes of abnormal liver biochemical tests. This list of differential diagnoses is broad and, together, accounts for more cases of liver enzyme disturbance in patients treated with ICIs than ILICI per se [22]. A non-exhaustive list is presented in Table 3. The presence of liver-specific autoantibodies and elevated immunoglobulin G (IgG) levels in peripheral blood are very rarely seen in ILICI and thus can be useful to distinguish ILICI from autoimmune hepatitis [50, 57]. Imaging in the form of ultrasound, computed tomography, or magnetic resonance imaging is required to assess structural anatomy, liver perfusion and to exclude other causes of pancreatobiliary pathology.

**Table 3:** Differential diagnosis for ILICI in the setting of liver biochemistry derangements

**Pattern of liver injury**	**Differentials**
Hepatocellular	Disease progression including metastatic disease, hepatitis A virus (HAV), hepatitis B virus (HBV), hepatitis C virus (HCV), hepatitis D virus (HDV), hepatitis E virus (HEV), Epstein Barr virus (EBV), cytomegalovirus (CMV), adenovirus, herpes simplex virus (HSV) types 1 and 2, varicella zoster virus (VZV), human immunodeficiency virus (HIV), chronic autoimmune hepatitis, alpha-1 antitrypsin deficiency, haemochromatosis, Wilson’s disease, other systemic illnesses or infections, alcohol use and other causes of drug induced liver injury.
Cholestatic	Disease progression, infection (e.g., bacterial cholangitis, pyogenic liver abscess), obstruction (malignant or non-malignant aetiology such as gallstone disease), primary biliary cirrhosis, primary sclerosing cholangitis, alpha-1 antitrypsin deficiency, IgG4 disease, bile duct stricture, thromboembolic disease, secondary cholangitis, alcohol use, other systemic illnesses or infections, other causes of drug induced liver injury.

ILICI: immune-mediated liver injury caused by immune checkpoint inhibitors; IgG4: immunoglobulin G4

Due to its invasive nature, liver biopsy is generally reserved for cases with atypical clinical and/or biochemical features, worsening liver biochemistry despite conventional ILICI management, or suspicion of pre-existing liver disease [50, 52, 58].

As in hepatocellular-type ILICI, the cholestatic form can occur asymptomatically, although patients may present with jaundice, especially in the context of sclerosing cholangitis [55]. Pyogenic liver abscesses have been reported as a complication of cholestatic-type ILICI related to sclerosing cholangitis [59]. The exclusion of other causes of cholestatic liver derangement is required for accurate diagnosis, as listed in Table 3. These include biliary obstruction, autoimmune cholestatic liver diseases such as primary biliary cirrhosis and primary sclerosing cholangitis, hepatobiliary manifestations of IgG4 disease, and other causes of drug-induced liver injury. Imaging is required to determine if there is large bile duct disease. Features that may be seen on imaging include extrahepatic bile duct dilatation and diffuse hypertrophy of the bile duct walls [60]. Small bile duct involvement is diagnosed histologically.

## Management

Management of patients with ILICI depends on the severity of liver injury and can be broadly stratified into (i) careful monitoring with continuation of ICI therapy, (ii) suspension of ICI therapy and commencement of treatment with corticosteroids, and (iii) institution of additional immunosuppression for corticosteroid-refractory cases.

In asymptomatic patients with mild elevation of liver enzymes consistent with grade 1 injury, it is generally considered that immunotherapy can safely be continued. It is nonetheless imperative that patients are monitored closely for biochemical progression and that other potential hepatotoxins, including alcohol consumption, are avoided.

In patients with grade 2 or above hepatotoxicity, ICIs should be withheld and corticosteroids should be administered. Suggested dosing regimens range from 0.5–2 mg/kg daily of prednisolone or equivalent [61, 62]. In cases of grades 3 or 4 ILICI, published guidelines of the American Society of Clinical Oncology, the European Society for Medical Oncology, and the American Gastroenterological Association recommend the use of high doses of methylprednisolone equivalents (1–2 mg/kg daily) with a taper to oral glucocorticoids once liver biochemistry demonstrates improvement [63–65]. It should be emphasised that these recommendations are based on expert consensus and that data available to properly inform the most appropriate corticosteroid dosing regimen are limited, especially in view of the well-known systemic side effects of corticosteroids and the potential adverse effect of corticosteroid treatment on cancer outcomes in patients treated with ICIs [62, 66]. A recent multi-centre retrospective cohort study of patients with grades 3 or 4 ILICI found that treatment with 1.5 mg/kg methylprednisolone daily or more was not associated with a more rapid rate of ALT improvement compared to patients treated with less than 1.5 mg/kg methylprednisolone daily but was associated with a higher rate of hyperglycaemia requiring treatment [62].

The optimal tapering regimen for corticosteroids is similarly not well established, although in practice this often involves steadily weaning over the course of 6 to 10 weeks, depending on the rates of improvement in serial liver biochemical tests. Recent studies have trialled more rapid initial weaning from higher daily doses, followed by a slow taper at a lower daily dose [67]. Notably, over 11% of ICI-treated patients who require corticosteroids for ILICI have been reported to develop an infection during their treatment course, most commonly pneumonia [62].

While corticosteroids are regarded as the current first-line standard of care for ILICI, some studies demonstrate spontaneous improvement in liver biochemistry in patients even with grades 3 or 4 liver enzyme derangements without the use of corticosteroids [51, 68, 69]. Given that such instances seemed truly reflective of ILICI rather than a non-ILICI-related intercurrent process, such reports raise the question of whether corticosteroids are necessarily required in all cases of ILICI. At present, there are no established predictors of response to corticosteroids. There are case reports describing the effective use of budesonide, a synthetic glucocorticoid with a high first-pass metabolism rate with minimal systemic absorption, potentially resulting in fewer side effects than traditional glucocorticoids, as an alternative to prednisolone for the management of ILICI [70, 71].

Patients who do not demonstrate improvement of liver biochemistry despite corticosteroids after 3 to 5 days are considered to have corticosteroid-refractory ILICI and proceed to management with second-line immunosuppression [63–65, 72]. Treatment approaches in these circumstances are modelled on immunosuppressive agents required for the treatment of autoimmune hepatitis and to prevent rejection following liver transplantation.

In instances of corticosteroid-refractory ILICI, mycophenolate mofetil is usually added, with doses ranging between 500 mg twice a day and 1,000 mg twice a day. Studies suggest that the addition of mycophenolate mofetil in these refractory cases may be more effective in improving liver biochemistry than continuing corticosteroids alone [61, 73]. Other immunosuppressive agents have also been used in the treatment of corticosteroid-refractory ILICI, often in the setting of failure to respond to mycophenolate mofetil as well, with evidence limited largely to case reports. These options include azathioprine, tacrolimus, cyclosporin, antithymocyte globulin, tofacitinib, tocilizumab, and plasma exchange [15, 61, 63, 74–79]. In particular, antithymocyte globulin has been effectively used in fulminant ILICI and should be considered in severe cases [72]. Infliximab has also been trialled but is predominantly used for the management of concurrent ICI-related colitis and the American Gastroenterological Association recommends that this agent should be used with caution in ILICI [61, 72, 80].

In patients who develop cholestatic-type ILICI, the response to immunosuppressive therapy is less well characterised, but available evidence suggests that this cohort responds relatively poorly to corticosteroids, with a corticosteroid response rate in the order of only 11% [41, 55, 81, 82]. Mycophenolate mofetil, tocilizumab, and azathioprine have been reported to be effective in small case series and case reports [83–87]. There are reports of ursodeoxycholic acid being used as an adjunct therapy to improve liver biochemistry and promote bile duct recovery, as in other forms of cholestatic liver injury, although the precise effect of ursodeoxycholic acid on the clinical trajectory of cholestatic ILICI remains to be fully determined [55, 88–90].

## Recommencement of ICI therapy after suspension for ILICI

ILICI is generally an acute process that does not progress to chronic liver injury with effective initial management [91]. ICIs are often safe to be recommenced once ILICI has improved [92, 93]. Recommencement of ICIs in patients with grade 2 ILICI can be considered once liver biochemistry has improved to grade 1 or less and corticosteroids have been tapered to doses such as prednisolone 10 mg once daily or less.

Societal guidelines have suggested the consideration of permanent discontinuation of ICIs in cases of grades 3 or 4 ILICI [64, 94]. Conversely, in a retrospective study of 31 melanoma patients with grades 3 or 4 ILICI who underwent a rechallenge with ICIs, only six patients (32%) experienced ICI adverse events requiring discontinuation, with four patients (13%) needing to discontinue therapy due to ILICI [95]. A similar safety signal has been reported in patients with HCC, in whom those with grades 3 ILICI were successfully rechallenged once they had resolution of liver biochemistry to grade 1 or less without recurrence of ILICI [96]. There is a tendency to rechallenge patients initially on an anti-CTLA-4 agent with an anti-PD-1 or anti-PD-L1 agent and to switch those initially on an anti-PD-1 or anti-PD-L1 agent to a different drug within these classes [95].

## Other hepatobiliary complications

### Hepatitis B virus (HBV) and hepatitis C virus (HCV) infection

Patients with active HBV and HCV infection were excluded from early ICI trials, but there has since been subsequent real-world evidence that ICIs are safe to use in this population with close monitoring [97–100].

Two large meta-analyses found that the incidence of HBV reactivation following treatment with ICIs was 1–1.3% in hepatitis B surface antigen (HBsAg) positive patients and 0% in HBsAg negative patients [40, 101, 102]. Notably, a ten-fold increase in the rate of HBV reactivation has been reported in those patients with chronic HBV infection not concurrently treated with antivirals compared to those who were [101]. This suggests that patients with chronic HBV infection should be commenced on antiviral therapy prior to initiating ICIs, regardless of viral load [103]. Rare instances of hepatitis B seroclearance and seroreversion during ICI therapy have been reported [104]. There have been no reported cases of HCV reactivation in patients treated with ICIs [100, 105].

### Prognosis

The prognosis related to ILICI depends on the severity of liver injury. Prognosis is favourable in grades 1 or 2 hepatocellular-type or corticosteroid-responsive ILICI cases. In a large multi-centre prospective trial, a higher incidence of liver enzyme disturbance attributed by the investigators to ILICI in an HCC cohort did not correlate to an increased need for corticosteroid treatment nor an increased rate of treatment discontinuation [31]. Time to normalisation of liver biochemistry can vary significantly, with studies demonstrating median time frames spanning weeks to months, including patients who responded to ICI suspension as well as corticosteroid-responsive patients [61, 68, 74, 106].

Specifically, regarding patients experiencing a cholestatic-type variant of ILICI, the time to normalisation of liver biochemistry can be prolonged, despite prompt ICI discontinuation and commencement of corticosteroids and other immunosuppressive agents as necessary [41, 106]. The less favourable natural history of cholestatic-type ILICI as compared to hepatocellular-type ILICI often results in the need to suspend ICI therapy for a prolonged period, predisposing to the progression of underlying cancer and thus an overall poorer prognosis [106].

The possibility that prognosis in patients with corticosteroid-refractory ILICI may be impaired consequent to an adverse effect of combination immunosuppressants in reducing the anti-tumour effect of ICIs and thereby promoting tumour progression in comparison to corticosteroids alone, as suggested in two recent reports [41, 107], is an important concept that will require further clarification from additional real-world data.

## Conclusions

With the increasing use of ICI therapy, including in patients previously excluded from clinical trials, awareness and understanding of ICI-related adverse events leading to best practice management of these are crucial. ILICI is not an uncommon ICI-related organ-specific adverse event but often has an insidious presentation. Routine liver biochemical test monitoring is essential to detect early stages of ILICI. Diagnosis is made through a process of exclusion of other causes of abnormal liver biochemical tests and careful assessment of liver imaging. Management depends on the grade of liver injury, but often involves a combination of ICI cessation and treatment with corticosteroids, with implementation of alternative immunosuppressive approaches in corticosteroid-refractory cases. In some instances, mild ILICI is transient and does not require intervention other than close surveillance. Recommencement of ICI treatments can be considered once mild grades of ILICI have improved or resolved, but those with grades 3 or 4 ILICI may require permanent discontinuation. Additional studies are required to further define the pathogenesis and natural history of ILICI, including both hepatocellular and cholestatic variants, in order to standardise best practice guidelines, including optimal dosing and weaning regimens of corticosteroid therapy and approaches to management in corticosteroid-refractory cases.

## Abbreviations

ALP: alkaline phosphatase
ALT: alanine aminotransferase
AST: aspartate aminotransferase
CTLA-4: cytotoxic T-lymphocyte-associated protein 4
HBV: hepatitis B virus
HCC: hepatocellular carcinoma
HCV: hepatitis C virus
ICI: immune checkpoint inhibitor
IgG: immunoglobulin G
ILICI: immune mediated liver injury caused by immune checkpoint inhibitors
PD-1: programmed death-1
PD-L1: programmed death ligand-1

## Declarations

### Copyright

© The Author(s) 2024.
